# Removal of Cr(VI) from Wastewater Using Acrylonitrile Grafted Cellulose Extracted from Sugarcane Bagasse

**DOI:** 10.3390/molecules29102207

**Published:** 2024-05-08

**Authors:** Idrees Khan, Ashraf Ali, Alia Naz, Zenab Tariq Baig, Wisal Shah, Zia Ur Rahman, Tawaf Ali Shah, Kotb A. Attia, Arif Ahmed Mohammed, Yaser M. Hafez

**Affiliations:** 1 Department of Environmental Science, Faculty of Physical & Applied Sciences, The University of Haripur, Haripur 22620, Pakistan; khanamazawal@gmail.com (I.K.); zenab.tariq@uoh.edu.pk (Z.T.B.); wisal@uoh.edu.pk (W.S.); zia@uoh.edu.pk (Z.U.R.); 2 School of Chemistry & Chemical Engineering, Henan University of Technology, Zhengzhou 450001, China; 3 Department of Chemistry, Faculty of Physical & Applied Sciences, The University of Haripur, Haripur 22620, Pakistan; 4 College of Agriculture Engineering and Food Sciences, Shandong University of Technology, Zibo 255000, China; tawafbiotech@yahoo.com; 5 Department of Biochemistry, College of Science, King Saud University, P.O. Box 2455, Riyadh 11451, Saudi Arabia; kattia1.c@ksu.edu.sa (K.A.A.); moaahmed@ksu.edu.sa (A.A.M.); 6 EPCRS Excellence Center, Plant Pathology and Biotechnology Laboratory, Agricultural Botany Department, Faculty of Agriculture, Kafrelsheikh University, Kafr El-Sheikh 33516, Egypt; hafezyasser@gmail.com

**Keywords:** cellulose, bagasse, grafting co-polymerization, water treatment, chromium

## Abstract

A highly efficient low-cost adsorbent was prepared using raw and chemically modified cellulose isolated from sugarcane bagasse for decontamination of Cr(VI) from wastewater. First, cellulose pulp was isolated from sugarcane bagasse by subjecting it to acid hydrolysis, alkaline hydrolysis and bleaching with sodium chlorate (NaClO_3_). Then, the bleached cellulose pulp was chemically modified with acrylonitrile monomer in the presence Fenton’s reagent (Fe^+2^/H_2_O_2_) to carry out grafting of acrylonitrile onto cellulose by atom transfer radical polymerization. The developed adsorbent (acrylonitrile grafted cellulose) was analyzed by X-ray diffraction analysis (XRD), scanning electron microscopy (SEM) and Fourier transform infrared spectroscopy (FT-IR). Both raw cellulose and acrylonitrile grafted cellulose were used for chromium removal from wastewater. The effects of metal ion concentration, pH, adsorbent dose and time were studied, and their values were optimized. The optimum conditions for the adsorption of Cr(VI) onto raw and chemically modified cellulose were: metal ion concentration: 50 ppm, adsorbent dose: 1 g, pH: 6, and time: 60 min. The maximum efficiencies of 73% and 94% and adsorption capacities of 125.95 mg/g and 267.93 mg/g were achieved for raw and acrylonitrile grafted cellulose, respectively. High removal efficiency was achieved, owing to high surface area of 79.92 m^2^/g and functional active binding cites on grafted cellulose. Isotherm and kinetics studies show that the experimental data were fully fitted by the Freundlich isotherm model and pseudo first-order model. The adsorbent (acrylonitrile grafted cellulose) was regenerated using three different types of regenerating reagents and reused thirty times, and there was negligible decrease (19%) in removal efficiency after using it for 30 times. Hence, it is anticipated that acrylonitrile could be utilized as potential candidate material for commercial scale Cr(VI) removal from wastewater.

## 1. Introduction

Increase in human population and rapid industrialization have led to the addition of various pollutants into the environment which affect living organisms [1]. Heavy metals (HMs) are added to the environment by different industries such as fertilizers production, pesticides production, untreated sewage, and petrochemical industry [2,3]. Even small amounts of heavy metals are harmful and cause anemia, damage to kidneys, intestines, liver, and can even lead to cancer [4,5,6]. The most toxic HMs are chromium, lead, mercury, cadmium, and arsenic, which cause several disorders in humans and animals [7,8,9,10]. Various industries, such as electroplating and the leather, textile, and tanning industries discharge chromium into the environment [11,12,13,14]. Chromium exists in nature in different oxidation states, among which the oxidation states of three Cr(III) and six Cr(VI) are more common. Cr(VI) is more toxic than Cr(III) and produces numerous infections such as malignant lung disease, chronic respiratory disease, irascibility and liver damage [15,16].

Various methods could be used for chromium removal, such as coagulation, membrane separation, filtration, electrocoagulation, reverse osmosis, electrodialysis, ultrafiltration, chemical precipitation and adsorption [17,18,19,20,21,22,23,24,25]. Most of these approaches are not useful, owing to lower removal efficiency, high energy consumption, high operational cost, the use of large quantities of chemicals and the production of secondary waste [20,26,27]. Adsorption is a more useful method because of high removal efficiency, low operational cost and simple operational design [28,29,30,31]. Different adsorbents are used for the removal of HMs, such as activated carbon, porous materials, ion exchangers, etc., but the high cost of commercial adsorbents is an obstacle in its use for wastewater treatment. Agriculturally based materials like fruit peels [32], rice husks [33], sawdust [34], bagasse [35], sugar beets, and soybean hull [36,37,38] have also been utilized for decontamination of HMs. However, agricultural by-product-based adsorbents have lignin and pectin, which cover the porous structure of cellulose and decrease its adsorption capacity. To tackle this challenge, the lignin and pectin can be removed by treating these materials with acid/alkali, followed by bleaching [39,40]. The cellulose fibers thus obtained are free from viscous and peptic compounds and have hydroxy, carbonyl, amine, etc. groups which can bind metal ions by electrostatic force of attraction or intermolecular force of attraction [41,42,43].

Chemical modification of cellulose fibers with a suitable ligand can enhance their interaction with HM ions, thus increasing their removal efficiency [44,45,46,47]. Therefore, it is necessary to modify natural cellulosic materials to improve their adsorption efficiency and their propensity to bind heavy metals [48]. Natural cellulosic materials have a high content of hydrogen bonding within the cellulosic chains, which prevents their interaction with metal ions [48,49]. Grafted cellulose has additional side chains which can bind the adsorbate molecules and thus the removal efficiency of grafted cellulose is higher than that of non-grafted or raw cellulose [50,51]. Several researchers have used sugarcane bagasse as an adsorbent, either in raw form or chemically modified form, for the removal of heavy metals, dyes and other pollutants. Ezeonuegbu et al. [52] utilized sugarcane bagasse for the removal of Pb(II) and Ni(II) from wastewater and obtained removal efficiencies of 89.31% and 96.33% for lead and nickel, respectively. Gusmano et al. [53] utilized modified sugarcane bagasse for the removal of etherdiamine from aqueous solution, and adsorption capacities of 869.6 and 1203.5 mg/g were obtained for succinic anhydride modified sugarcane bagasse and EDTA dianhydride modified sugarcane bagasse, respectively. In another study, Al-Mokhalelati et al. [54] used chemically modified sugarcane bagasse for the removal of methylene blue from aqueous solutions and obtained 98% removal efficiency for MB. Similarly, Jiang et al. [55], utilized sugarcane bagasse modified with polyethylenimine for the removal of Cu(II) from aqueous solution and adsorption capacities up to 107.5 mg/g were reported. The removal efficiency of raw sugarcane bagasse is not high enough to remove heavy metal ions, dyes or other organic pollutants at low concentration, owing to the presence of several other compounds such as pectin, lignin, etc., in addition to cellulose. To remove these peptic compounds and enhance the removal efficiency of sugarcane bagasse, it was subjected to acid hydrolysis, alkaline hydrolysis, and bleaching. After hydrolysis and bleaching, clean white cellulose fibers were obtained, which were functionalized with acrylonitrile to incorporate a nitrile group. The insertion of the nitrile group further enhances the interaction of cellulose with heavy metals, dyes and other pollutants; therefore, chemical modification of cellulose was carried out.

## 2. Results and Discussion

### 2.1. Characterization

#### 2.1.1. Surface Morphology

**Figure 1:**
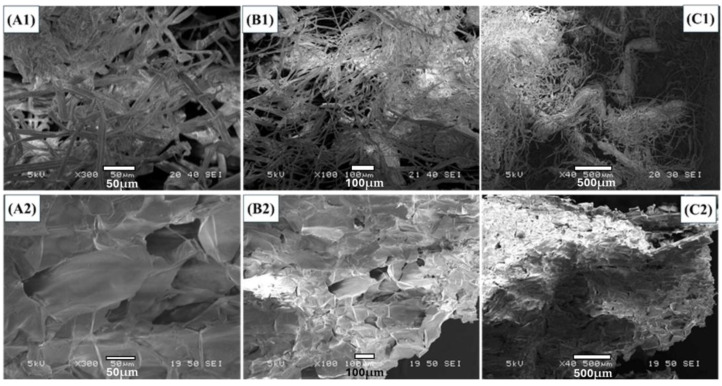
SEM images of raw sugarcane bagasse (**A1**,**B1**,**C1**), and grafted sugarcane bagasse (**A2**,**B2**,**C2**) in different resolutions.

Figure 1 shows the SEM images of raw sugarcane bagasse and acrylonitrile grafted cellulose (GC) isolated from sugarcane bagasse at different magnifications. The SEM images of raw sugarcane bagasse (Figure 1(A1,B1,C1)) show a cluster of fibers that are stuck together, which could be owing to the presence of viscous substances (lignin & pectin). The SEM images in Figure 1(A2,B2,C2) show that after chemical treatment and grafting, the surface morphology of sugarcane bagasse has been changed extensively. The change in surface morphology of sugarcane bagasse after hydrolysis and bleaching is owing to the removal of peptic compounds and lignin, and the breaking down of long cellulose fibers into shorter ones. The SEM images in Figure 1(A2,B2,C2) show that the morphology of acrylonitrile grafted sugarcane bagasse is smoother than the raw bagasse, owing to chemical treatment and the attachment of nitrile monomer onto the cellulose surface. Similar changes in the surface morphology of cellulosic adsorbents were reported by Ali et al. [51].

#### 2.1.2. XRD Analysis

**Figure 2:**
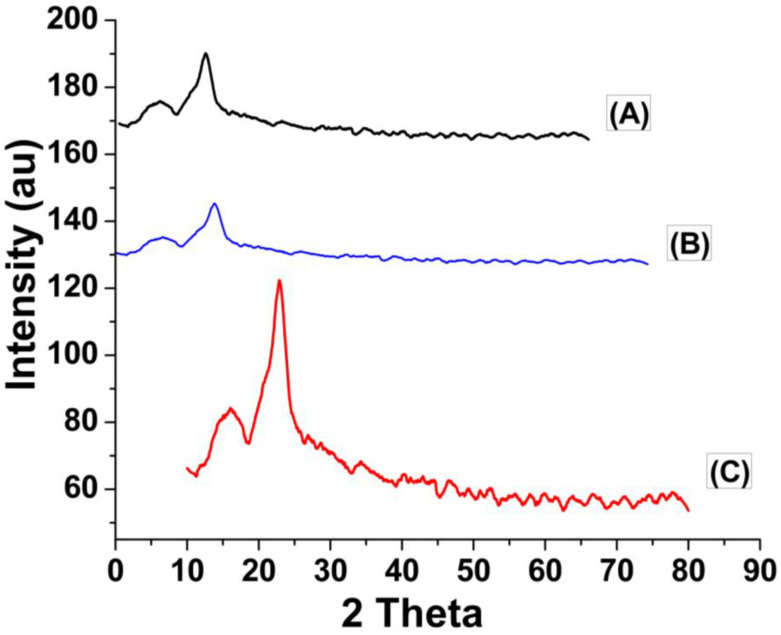
XRD analysis of unmodified cellulose isolated from SB (**A**), grafted cellulose isolated from SB before adsorption (**B**) and grafted cellulose isolated from SB after Cr(VI) adsorption (**C**).

Figure 2 shows the X-rays diffraction results of unmodified cellulose (A) and acrylonitrile grafted cellulose before adsorption (B) and after adsorption (C). In Figure 2, the XRD spectra of unmodified cellulose (A) and grafted cellulose before adsorption (B) show only small peaks at 13° and 13.2°, respectively, while the rest of the spectrum has no prominent peak in the range of 20–25°, as can be seen in Figure 2A,B. The results verify that following surface functionalization, the cellulose’s crystallinity index (CrI) remains unchanged. The XRD spectra of grafted cellulose (Figure 2B) and unmodified cellulose (Figure 2A) showed no discernible variation in the XRD spectrum width. The primary crystalline cellulose I peak remained unaltered during grafting reactions, according to the XRD data. However, upon Cr(VI) adsorption, the XRD spectrum of the grafted cellulose showed noticeably wider widths than those of the ungrafted samples and had a sharp peak at 22.72° at 2θ value, indicating that Cr(VI) ions adsorbed onto GSB (Figure 2C). The XRD result of GSB confirms Cr(VI) adsorption on GSB, and the peaks at 22.72° correspond to Cr_2_O_3_ having a tetragonal crystalline phase, according to the inorganic crystalline library [56,57].

#### 2.1.3. FTIR Analysis

**Figure 3:**
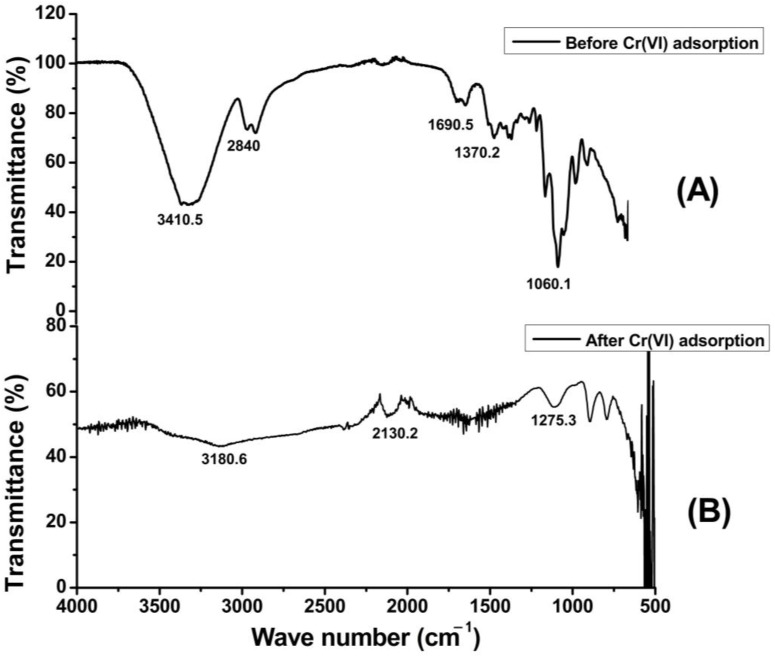
FT-IR analysis of unmodified cellulose isolated from sugarcane bagasse (**A**) and chemically modified cellulose isolated from sugarcane bagasse (**B**).

The FTIR analysis results of unmodified cellulose (UGC) and grafted cellulose isolated from sugarcane bagasse (GC) are presented in Figure 3A and B, respectively. The FTIR spectrum in Figure 3A shows that the peaks at 2870 cm^−1^ and 3391cm^−1^ are representing the C–H and O–H bond stretching vibration frequencies, respectively. In Figure 3B, which represents acrylonitrile grafted cellulose, the intensities of these peaks are very low, which shows that most of these groups have been modified with a CN group after grafting co-polymerization. Similarly, a prominent peak at 1656 cm^−1^ in Figure 3B accounts for the nitrile (–CN) group and confirms the insertion of a nitrile group onto the surface of the cellulose. There are some other minor peaks at 1690 cm^−1^ which represent the vibration frequency of water molecules (Figure 3A). In Figure 3B, another peak can be seen at 1510 cm^−1^, which represents the carboxyl group stretching vibration [41], while this peak does not exist in raw sugarcane bagasse [42].

### 2.2. Effect of Various Parameters on Cr(VI) Removal

#### Effect of Metal Ion Concentration

**Figure 4:**
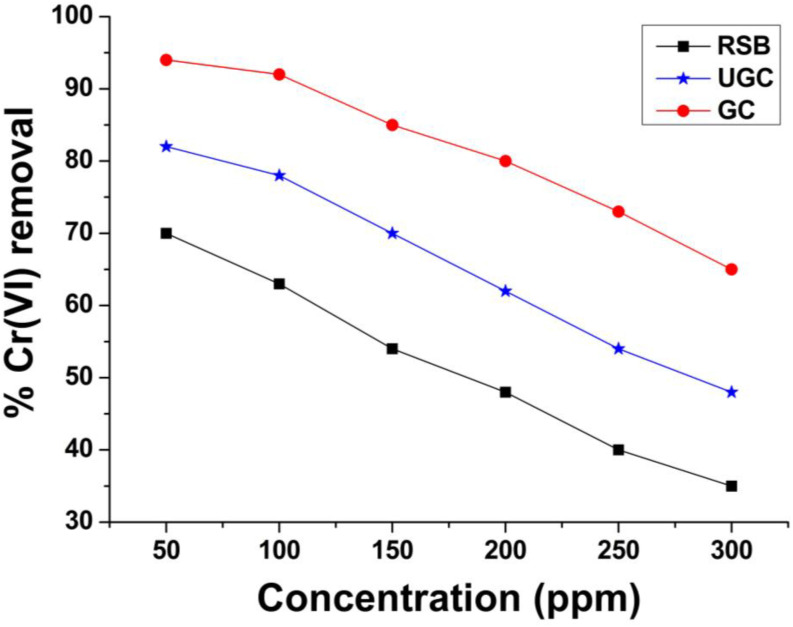
Effect of HM concentration on the removal efficiency of Cr(VI) by raw sugarcane bagasse (RSB), un-grafted cellulose (UGC) and grafted cellulose (GC) extracted from sugarcane bagasse.

The adsorption efficiency depends upon the concentration of adsorbate ions/molecules in a medium. The accumulation of analyte molecules/ions onto the surface of an adsorbent depends upon the available surface area and the number of active sites or functional groups. To explore the metal ion concentration effect on the removal efficiency of raw, unmodified and grafted sugarcane bagasse, Cr(VI) solutions of 50–300 ppm were checked during batch experiments while adsorbent dose, pH, time and temperature were held constant. In flasks, 100 mL chromium solutions of 50, 100, 150, 200, 250 and 300 ppm were taken, each with 1 g of adsorbent and pivoted at 300 rpm for 2h at 25 °C. After 2 h filtration was carried out to remove the adsorbent, the C(VI) ion concentration was determined by AAS. The same procedure was performed for raw sugarcane bagasse. Figure 4 shows that 70%, 82% and 94% removal efficiencies were obtained for raw sugarcane bagasse (RSB), un-grafted cellulose obtained from sugarcane bagasse (UGC) and grafted cellulose (GC), respectively, at lower concentrations, i.e., 50 ppm, and the removal efficiency decreased with increasing concentration. The removal efficiency of adsorbents declined with the increase in concentration and reached 35%, 48% and 65% at concentrations of 300 ppm for RSB, UGC and GC, respectively. Active sites on the adsorbent were sufficient to bind the metal ions when the concentration was low. By raising the concentration of HMs above the optimum values, then the adsorbent cannot adsorb more molecules, and thus, the removal efficiency decreases. The removal efficiency decreases at high concentrations owing to the saturation of active sites with metal ions, and no further ions can be picked up by adsorbents. The removal efficiency of GSB is higher than un-grafted cellulose (UGC) and raw sugarcane bagasse (RSB) at both lower and higher concentrations. The GC has 94% and 65% removal efficiency at 50 ppm and 300 ppm, respectively, while the removal efficiency of unmodified cellulose (UGC) is 82% and 48%, and for RSB, 70% and 35% at 50 ppm and 300 ppm concentration, respectively, which indicates the superior performance of GC over UGC and RSB. The finding of the current study matches well with other similar studies [46,47].

### 2.3. Effect of Adsorbent Mass

**Figure 5:**
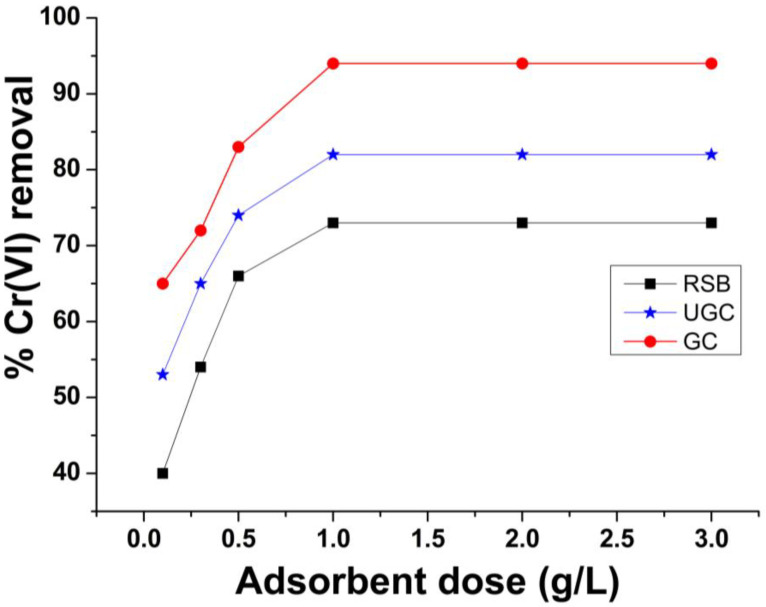
Effect of adsorbent mass (g/L) on Cr(VI) removal by raw sugarcane bagasse (RSB), un-grafted cellulose (UGC) and grafted cellulose (GC) extracted from sugarcane bagasse.

In batch experiments, different quantities (0.1, 0.3, 0.5, 1, 2 and 3 g) of raw sugarcane bagasse (RSB), un-grafted cellulose (UGC) and grafted cellulose (GC) were taken in separate flasks, and 100 mL Cr(VI) solution (50 ppm) was taken in flasks. The pH was maintained at 4, and the flasks were agitated at 300 rpm for 60 min and filtered, and Cr(VI) was measured by AAS. Figure 5 shows the relationship of the adsorbent dose and removal efficiency of RSB, UGC and GC. The results show that at 0.1 g adsorbent dose, removal efficiencies of 38%, 50 and 67% efficiency were obtained for RSB, UGC and GC, respectively. Figure 5 shows that Cr(VI) removal was enhanced with the increase in adsorbent mass from 0.1 g and reached maximum values of 70%, 81% and 94% for RSB, UGC and GC, respectively. Figure 5 shows that there was no change in % removal when the adsorbent quantity was increased beyond 1.0 g and a straight line was obtained. The removal efficiency was lower when the adsorbent quantity was lower than the optimum dose because the mass of adsorbent was inadequate for capturing metal ions, and thus, low removal efficiency was recorded for RSB, UGC and GC. Initially, the percentage removal efficiency (%R) was increased because the number of active sites was high enough for the capturing of Cr(VI) ions. By enhancing the adsorbent mass from 1 to 3 g, no further increase was observed in removal efficiency because the number of active sites of adsorbent at this quantity (1 g) was high enough for picking Cr(VI) ions (50 ppm concentration). A similar trend was observed in other studies on heavy metal removal [50].

### 2.4. Effect of Time

**Figure 6:**
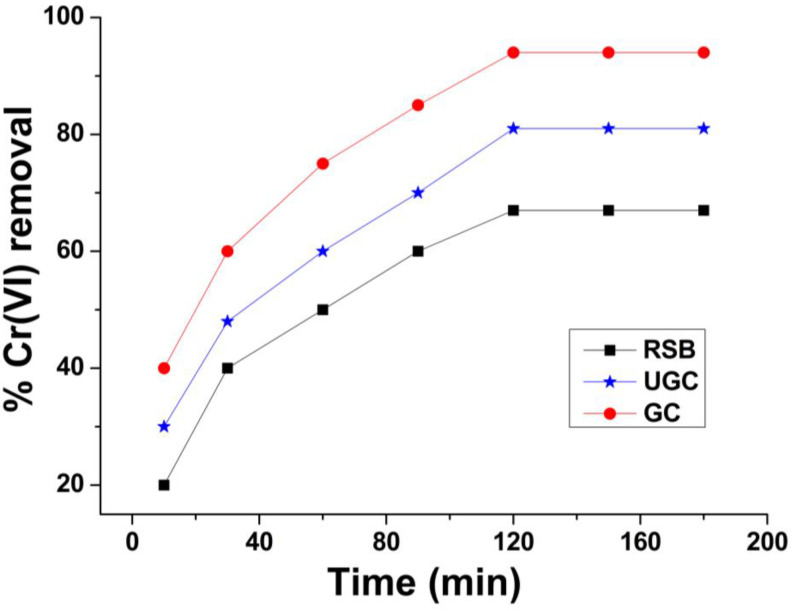
Effect of contact time on Cr(VI) adsorption onto raw sugarcane bagasse (RSB), un-grafted cellulose (UGC) and grafted cellulose extracted from sugarcane bagasse (GC).

The Cr(VI) removal by raw sugarcane bagasse (RSB), un-grafted cellulose (UGC) and grafted cellulose (GC) at different times is shown in Figure 6. Chromium solution (50 ppm) was taken in seven conical flasks (100 mL in each flask) and stirred for 10, 20, 30, 60, 120, 150 and 180 min for each adsorbent. Initially, when the adsorption time was increased, the Cr(VI) removal efficiency was increased, until 120 min. At 120 min, the removal efficiency reached maximum values of 67%, 81% and 94% for RSB, UGC and GC, respectively. Increasing adsorption time to 180 min does not change the removal efficiency, and the same efficiency was recorded because that adsorption equilibrium was achieved within 120 min. The removal efficiency was not enhanced beyond 120 min because the active sites of the adsorbent were occupied by metal ions within 120 min. The rate of metal ions removal was faster at the beginning owing to the large number of vacant active sites on the adsorbent [58].

### 2.5. Effect of pH on the Removal of Cr(VI)

**Figure 7:**
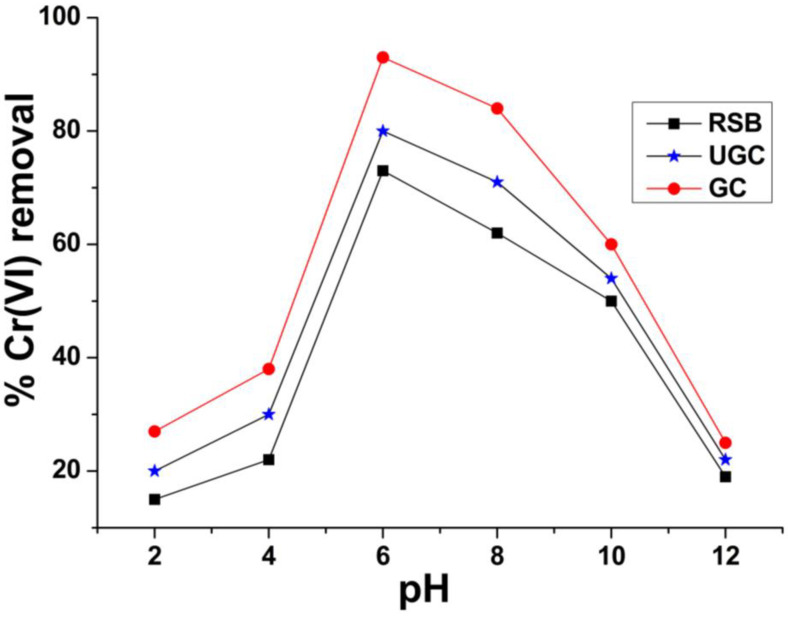
Effect of pH Cr(VI) removal by raw sugarcane bagasse (RSB), un-grafted cellulose (UGC) and grafted cellulose (GC) extracted from sugarcane bagasse.

Figure 7 shows the adsorption of Cr(VI) onto raw sugarcane bagasse (RSB), un-grafted cellulose (UGC) and grafted cellulose obtained from sugarcane bagasse (GC) at various pH values. The removal efficiency is low at pH 2–4, and increases sharply to a maximum value of 94% at pH 6. Further elevating the pH beyond 6 decreases the Cr(VI) removal, which reached a minimum value of 17 and 20% for raw and grafted sugarcane bagasse, respectively, at pH 12. At pH more than 6, the Cr(VI) removal decreased owing to OH^−^ ions present in the medium, which interact with adsorbent instead of CrO_2_^−2^ ions and occupy the positively charge sites on the adsorbents. Similarly, at pH below 6, abundant H^+^ ions interrupt the ability of Cr(VI) cations to adsorb over raw and GC, and thus, the efficiency of metal ion removal decreases [59].

### 2.6. Isotherm Study

Isotherm models show the process of adsorbate accumulation on the adsorbent’s surface and provide the information about the type of adsorption: chemical or physical, monolayer or multilayer, etc. [60]. The experimental data of Cr(VI) accumulation on raw sugarcane bagasse (RSB), un-grafted cellulose (UGC) and grafted cellulose (GC) isolated from sugarcane bagasse was computed by the Freundlich model and Langmuir model [61].

#### 2.6.1. Freundlich Model

The Freundlich isotherm model in linear form can be represented as given in Equation (1).

(1) $$\ln q=lnK\frac{1}{n}C_{e}$$

**Figure 8:**
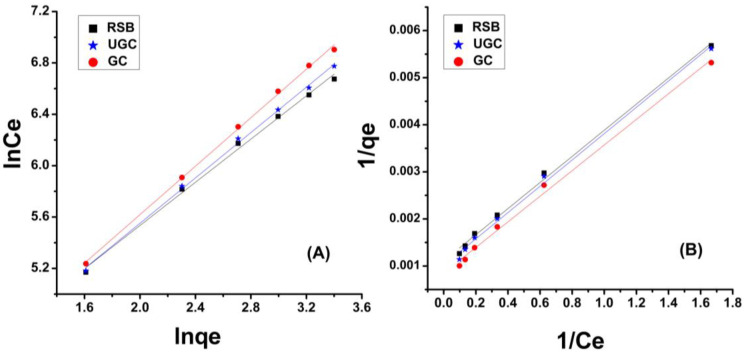
Freundlich (**A**) & Langmuir (**B**) isotherm model for Cr(VI) adsorption onto raw sugarcane bagasse (RSB), un-grafted cellulose (UGC) and grafted cellulose (GC) isolated from sugarcane bagasse.

According to this model, the adsorption takes place over a heterogeneous surface, forming multiple layers of adsorbate molecules, and increases with the increase in concentration of an adsorbate. The adsorption data of Cr(VI) adsorption onto RSB & GSB was computed by the Freundlich isotherm model. The numerical values of “q” and “C_e_” were calculated and lnq_e_ was plotted against lnC_e_. From the linear plot, the values of 1/n (slop), K (L mg^−1^) and “n” were calculated as given in Equation (1). The results in Figure 8A show that the adsorbents (RSB, UGC and GC) are effective for Cr(VI) removal [62]. The numerical values of “1/n”, KF and R^2^ obtained from Cr(VI) removal on RSB and GSB are presented in Table 1. The “1/n” values for RSB, UGC and GC are 42.45, 64.30 and 124.36, respectively. The values of the Freundlich constant (KF) of 2.821, 4.72 and 9.786 and R^2^ of 0.97, 0.97 and 0.98 were recorded as shown in Table 1.

#### 2.6.2. Langmuir Isotherm Model

This model predicts the homogeneous surface of adsorbent with a limited number of active sites, and the adsorption of adsorbate molecules/ions is monolayer. Equation (2) shows the mathematical form of the Langmuir isotherm model.

(2) $$\frac{C_{e}}{q}=\frac{1}{q_{max}}K+\frac{C_{e}}{q_{max}}$$

where “C_e_” is the equilibrium concentration and “q_e_” is the quantity of adsorbate adsorbed per unit mass of adsorbent (g). The adsorption capacity is represented by “q_max_ (mg g^−1^)” and KL (L mg g^−1^) is the constant of adsorption energy [24]. The q_max_, K and KL (L mg g^−1^) values were calculated from C_e_/q versus C_e_ plot and linear regression. The values of “q_max_”, KL, R^2^ and (RL) were calculated using Equation (3) and a plot of 1/C_e_ vs. 1/q_e_ as shown in Figure 8B.

(3) $$RL=\frac{1}{\left(1+KLC_{0}\right)}$$

The values of “q_max_”, KL and R^2^ were found by plotting 1/C_e_ vs. 1/q_e_ and the data are given in Table 1. The numerical values of q_max_ were 125.95 mg/g, 202.37 mg/g and 267.93 mg/g; of K (L/mg), 4.5808, 4.1733 and 3.9563; and of R^2^, 0.91, 0.89 and 0.84 for GSB, UGC and GC, respectively (Table 1). The results suggest that the adsorption of Cr(VI) onto RSB, UGC and GC fits more closely with the Freundlich model. The R^2^ of the Freundlich and Langmuir models for RSB, UGC and GC are given in Table 1, which indicates that the adsorption data of Cr(VI) is fitted will with the Freundlich isotherm model.

### 2.7. Adsorption Kinetics

The mechanism and rate-limiting step of reaction are determined from the kinetics study. In the current study, batch adsorption experiments were conducted with different times to find the optimum time where the equilibrium established and maximum adsorption of Cr(VI) takes place over RSB and GSB. The kinetics results show that Cr(VI) adsorption was slow at initial stages and reached equilibrium in 120 min, which indicates that the adsorption was chemisorption, and the adsorbate interacts with the active sites of the adsorbent by chemical interactions. Moreover, to find the rate-determining step, the data were computed by various kinetics models [63].

#### 2.7.1. Pseudo First-Order Model

The pseudo first-order kinetic model can be represented as

(4) $$\ln\left(q_{e}-q_{t}\right)=lnq_{e}-K_{1}t$$

**Figure 9:**
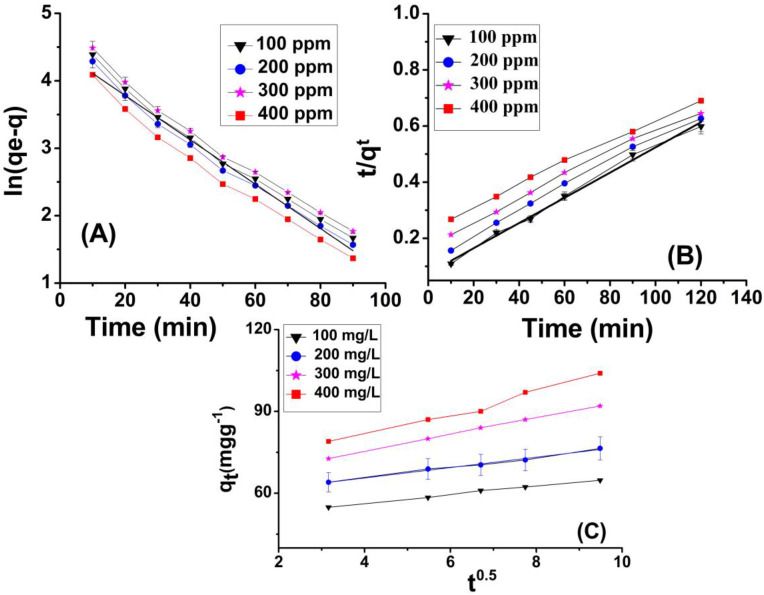
Pseudo first-order model (**A**), pseudo second-order model (**B**) and “intra-particle diffusion model” (**C**) for adsorption of Cr(VI) onto grafted cellulose (GC) isolated from sugarcane bagasse.

In Equation (4), “q_t_” and “q_e_” are the quantity of Cr(VI) ions adsorbed at specific time and equilibrium, respectively, while “K_1_” is the constant [60]. Cr(VI) solutions of 100, 200, 300 and 400 ppm were taken in different containers, each containing 0.4 g of adsorbent (GC), and the adsorption was checked from 10 min to 180 min. Adsorption capacity at a specific time (q_t_) and equilibrium time (q_e_), and rate constant K_1_ (min^−1^), were calculated from a (q_e_/q_t_) vs. (t) graph (Figure 9A).

#### 2.7.2. Pseudo Second-Order Model

Equation (5) shows the pseudo second-order kinetic model.

(5) $$\frac{1}{q_{t}}=\frac{1}{k_{2}q_{e}^{2}}+\frac{1}{q_{e}}$$

In Equation (5), “K_2_” is the rate constant and “q_t_” and “q_e_” are the quantity of Cr(VI) adsorbed at specific time (t) and equilibrium time, respectively. Cr(VI) solutions of 100, 200, 300 and 400 ppm were taken in different flasks containing GSB and the adsorption was checked from 10 min to 180 min. The value of q_max_ at specific time (qt) and equilibrium time (q_e_), and K_2_ (gmg^−1^ min^−1^) were calculated (t/q_t_) vs. t plot [64].

#### 2.7.3. Intra-Particle Diffusion

HM ions from the mixture/medium transfer to adsorbent by intraparticle diffusion [65]. The mathematical form of the intra-particle diffusion model can be represented as Equation (6).

(6) $$q_{t}=K_{p}t0.5+C$$

where q_t_ (mgg^−1^) is Cr(VI) adsorbed at specific time (t), K_p_ (mgg^−1^ min^0.5^) is a constant (diffusion within the particle) and C is the point of intersection. During batch experiments, four standard solutions of Cr(VI) of specific concentration (100, 200, 300, 400 ppm) were prepared, and adsorption over GSB was carried out for different time intervals (10 min to 180 min). The adsorption capacity at a specific time (q_t_) was plotted against the square root of time (t0.5). A plot of (q_t_) vs. (t0.5) is given in Figure 9C [27]. The numerical values of different constants of all three models, as well as their regression square (R^2^), are shown in Table 2. The R^2^ value of the pseudo second-order model is 0.99, which the highest among all three models.

### 2.8. Regeneration and Reuse of Spent Adsorbent (GSB)

The spent adsorbent (GC) collected from batch Cr(VI) adsorption experiments was divided into three equal parts, each weighted 100 g, and each portion was treated with 0.1 M NaOH, 0.1 M H_2_SO_4_ and 1 M ethylenediaminetetraacetic acid (EDTA) solutions. In a typical experiment, 100 g of spent GSB was added into a container containing 700 mL of 1 M NaOH and stirred in a reciprocal shaker at 45 °C for 3 h, followed by filtration. The percent recovery of chromium (VI) ions was determined from Equation (7). A similar method was used for the desorption of chromium from spent adsorbent (GSB) using other desorbing reagents, such as sulfuric acid and ethylenediaminetetraacetic acid (EDTA) solutions.

% Cr(VI) desorption = (Mass of Cr(VI) desorbed (mg/g))/(Mass of Cr(VI) adsorbed (mg/g)) × 100(7)

**Figure 10:**
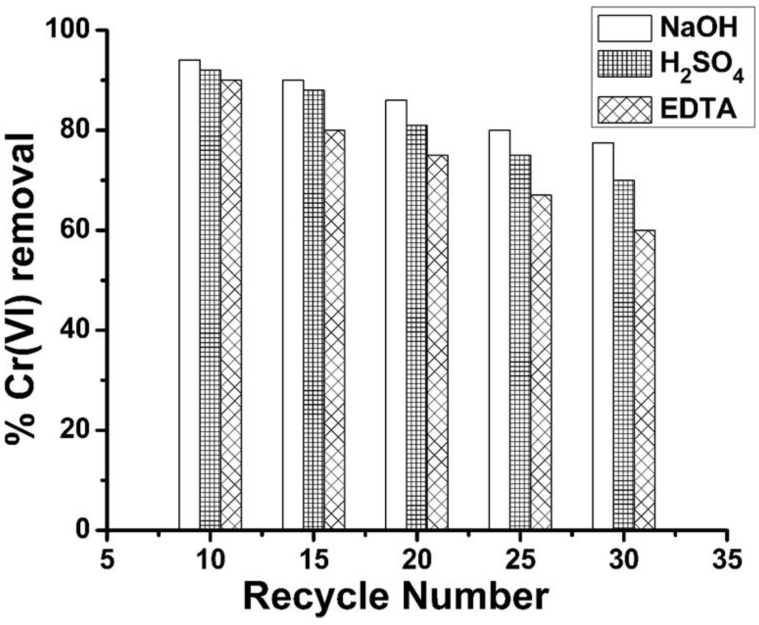
Average removal efficiency of spent adsorbent (GSB) after regeneration several times with different reagents.

The metal ion removal efficiency of regenerated GSB is presented in Figure 10. The maximum Cr(VI) removal efficiency (average of 85.5%) was obtained for NaOH-treated spent adsorbent (GC) after 30 cycles of regeneration and reuse, followed by H_2_SO_4_ (81.2%) and EDTA (74.4%) regenerated adsorbent. The results suggest that grafted sugarcane bagasse could be used many times after regeneration.

## 3. Material and Methods

### 3.1. Adsorbent Preparation

Sugarcane bagasse was taken from Haripur, Pakistan, washed and dried. Then the dried sugarcane bagasse was subjected to acid hydrolysis, alkali hydrolysis, bleaching and grafting with acrylonitrile [51]. The preparation of acrylonitrile grafted cellulose is discussed below in detail and the illustration of cellulose extraction from sugarcane bagasse is shown in Figure S1.

#### 3.1.1. Acid Hydrolysis

Crude sugarcane bagasse (200 g) was taken in a flask and 2% 1 M HCl (500 mL) was added into it. The contents were boiled for 3 h on a hotplate. After acid hydrolysis, the material was washed several times.

#### 3.1.2. Alkaline Hydrolysis

After acid hydrolysis, the sample was further hydrolyzed with sodium hydroxide solution. Acid-hydrolyzed sugarcane bagasse was heated with 2% NaOH solution for 3 h at 100 °C, followed by washing with distilled water until neutralized.

#### 3.1.3. Bleaching

The cellulose pulp obtained after alkaline hydrolysis was treated with sodium chlorate (0.1 mg) and glacial acetic acid (0.1 mL) at 40 °C. The reddish-brown color of cellulose pulp disappeared after bleaching, and the white pulp obtained after bleaching was dried and stored.

#### 3.1.4. Grafting Co-Polymerization

Bleached sugarcane bagasse (100 g) was placed in a conical flask; 0.5 g FeSO_4_, deionized water (200 mL), hydrogen peroxide (1 mL) and acrylonitrile (0.3 mL) were added into it and stirred at 40 °C for 1h, followed by washing with DW and drying. Soxhlet extraction of the acrylonitrile grafted cellulose pulp in anhydrous toluene was performed at 100 °C to eliminate the unreacted monomers.

### 3.2. Characterization

SEM provides information about the sorbent’s morphology and the surface area that can be used for adsorption. An FT-IR spectrometer (TENSOR II, Bruker, Bremen, Germany) was used to measure the functional groups. The crystallinity was confirmed by X-ray diffraction examination utilizing an DW-XRD-Y300 X-ray diffractometer (Chongqing, China).

### 3.3. Adsorption of Cr(VI) on RSB and GSB

A standard solution of Cr(VI) (1000 ppm) was diluted to 50, 100, 150, 200, 250 and 300 ppm for use in batch adsorption experiments. Cr(VI) solution (100 mL) from every dilution was taken in flasks, followed by the addition of 1 g adsorbent (RSB and GSB) and pivoted at 300 rpm for 60 min. In the filtrate, the Cr(VI) ion concentration was measured by ASS. Equation (8) was used to find out the adsorption capacity (q_e_).

(8) $$q_{e}=\frac{V\left(C_{0}-C_{e}\right)}{W}$$

where “q_e_” is the mass of heavy metals, “V” is the volume of the solution in liters, “W” is the mass of adsorbent in grams, C_0_ is the initial concentration of HMs and C_e_ is the equilibrium concentration. The % removal efficiency was found using Equation (9).

(9) $$Removal\;efficiency\;\left(\%\right)=\frac{\left(C_{0}-C_{e}\right)}{C_{0}}\times 100$$

### 3.4. Isotherms Study

To find the mechanism of interaction of adsorbate with adsorbent, and the surface heterogeneity of the adsorbent, the experimental data were computed by different isotherm models, such as Freundlich model and Langmuir model. The linear regression was performed using MS Excel 2020 and Origin pro 8.5 software.

### 3.5. Adsorption Kinetics

A kinetics study was performed in order to check the time at which equilibrium is established for Cr(VI) adsorption at different concentrations and to find the rate determining step [48]. The experimental data (q_e_, q_t_, etc.) obtained at different concentrations and different time were computed by first-order, second-order and intraparticle diffusion models [49].

## 4. Conclusions

In conclusion, the developed adsorbents raw sugarcane bagasse (RSB) and nitrile grafted sugarcane bagasse (GSB) can effectively remove Cr(VI) from wastewater. Cellulose fibers were isolated from sugarcane bagasse, which is a cheap, abundant agricultural byproduct. The removal efficiency of raw sugarcane bagasse, un-grafted cellulose and grafted cellulose isolated from sugarcane bagasse were 73%, 82% and 94%, respectively. The effects of concentration, pH, adsorbent amount, and time were checked, and the optimum values were determined. The adsorption data of Cr(VI) were fitted with the Freundlich isotherm model and follow the pseudo second-order kinetics model. The spent adsorbent was regenerated using different regenerating reagents such as sulfuric acid, sodium hydroxide and ethylenediamine tetra acetic acid, and reused for Cr(VI) ion removal from wastewater. The removal efficiency of the regenerated absorbent was 85.5% for NaOH-regenerated adsorbent, 81.2% for H_2_SO_4_-regenerated adsorbent and 74.4% for EDTA-regenerated adsorbent after thirty cycles. The high removal efficiency of both newly prepared and regenerated adsorbent (GC), as well as the low cost, suggest that it could be utilized as an efficient alternative adsorbent for the water treatment. In -future, GC will be utilized for the removal of other HMs and dyes to check its performance for the removal of other analytes.

## Figures and Tables

**Table 1:** Numerical values of Freundlich & Langmuir isotherm model for raw sugarcane bagasse (RSB), un-grafted cellulose (UGC) and grafted cellulose (GC) isolated from sugarcane bagasse.

Adsorbent	Freundlich Model			Langmuir Model		
	**1/n**	**K_F_ (mg/g)**	**R^2^**	**q_max_ (mg/g)**	**K (L/mg)**	**R^2^**
RSB	42.45	2.8210	0.97	125.95	4.5808	0.91
UGC	63.30	4.7237	0.97	202.37	4.1733	0.89
GC	124.36	9.7863	0.98	267.93	3.9563	0.84

**Table 2:** Pseudo first-order, pseudo second-order model and “intra-particle diffusion model” values for Cr(VI) adsorption onto GSB.

Concentration (ppm)	First-Order			Second-Order			IPD Model	
	q_e_ (mgg^−1^)	K_1_	R^2^	q_e_ (mgg^−1^)	K_2_	R^2^	R^2^	K_i_ (mgg^−1^min^−1^)
100	188	0.644	0.938	176	0.0137	0.94	0.9713	2.0828
200	368	0.66	0.978	336	0.0242	0.95	0.9005	2.4641
300	546	0.637	0.936	480	0.0451	0.97	0.9281	3.2259
400	720	0.681	0.989	592	0.0550	0.99	0.9172	4.0492

## Data Availability

The data are available from the corresponding author and may be provided upon written request.

## Supplementary Materials

The following supporting information can be downloaded at: https://www.mdpi.com/article/10.3390/molecules29102207/s1, Figure S1: Isolation of cellulose from sugarcane bagasse by acid hydrolysis, alkaline hydrolysis, bleaching and grafting.
