# Supplementary material for: Removal of Cr(VI) from Wastewater Using Acrylonitrile Grafted Cellulose Extracted from Sugarcane Bagasse
Source: Molecules. 2024 May 8;29(10):2207. doi: 10.3390/molecules29102207 (PMC11124459; doi:10.3390/molecules29102207)
Supplement: Supplementary file 1 [file molecules-29-02207-s001.zip › molecules-2940719-supplementary.pdf]

<Supplementary material>

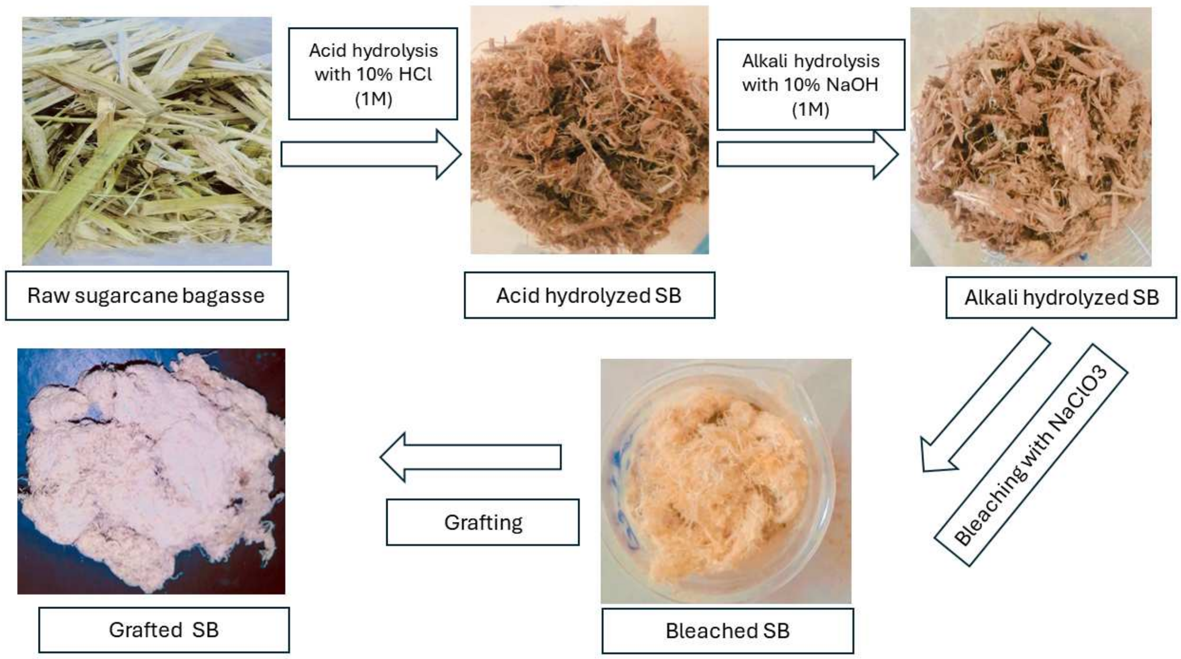

Figure S1. Isolation of cellulose from sugarcane bagasse by acid hydrolysis, alkaline hydrolysis, bleaching and its grafting.
